# Supplementary material for: PTP4A1 promotes TGFβ signaling and fibrosis in systemic sclerosis
Source: Nat Commun. 2017 Oct 20;8:1060. doi: 10.1038/s41467-017-01168-1 (PMC5651906; doi:10.1038/s41467-017-01168-1)

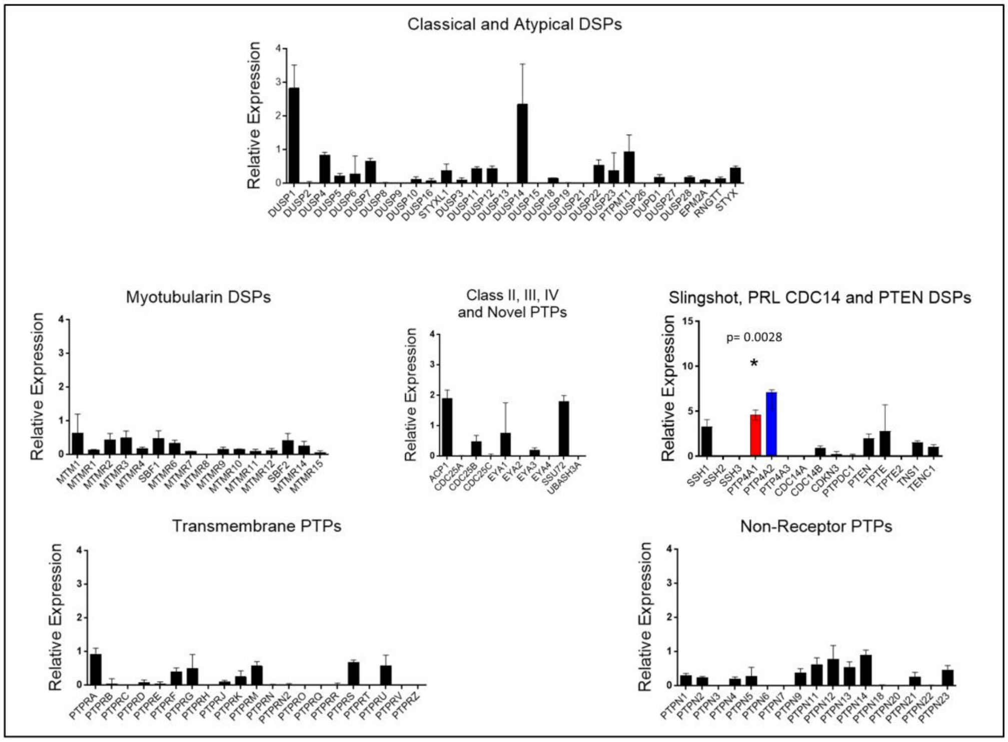

Supplementary Figure 1. PTPs expression in SSc dermal fibroblasts. mRNA expression of all 109 PTP genes was measured in 4 SSc human dermal fibroblast lines and compared to 5 healthy donors. *POLR2A* expression was used for normalization. The red and blue bars are representative of PTP4A1 and PTP4A2, respectively. *, $p<0.05$, Mann-Whitney test.

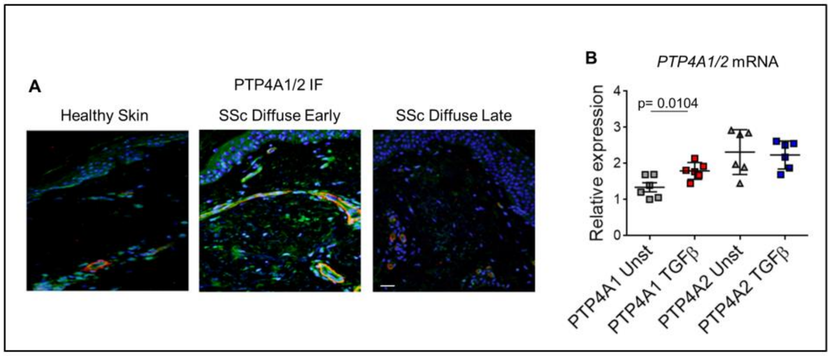

Supplementary Figure 2. PTP4A1 is overexpressed in SSc dermal fibroblasts. (**A**) PTP4A1/2 (green signal) and αSMA (red signal) immunofluorescence in skin sections from a second cohort of 5 healthy, 5 SSc early or 5 SSc late donors with Hoechst nuclear staining (blue signal). PTP4A1/2-αSMA co-localization signal appears orange in the images. Mean±SEM of PTP4A1/2 fluorescence densitometry signal in αSMA positive skin cells from this cohort is shown in **Fig. 1A**. (**B**) Mean±SEM of *PTP4A1* and *PTP4A2* mRNA relative expression measured in 6 different NHDF lines stimulated with TGFβ. Mann-Whitney test. Scale bar, 50 μm

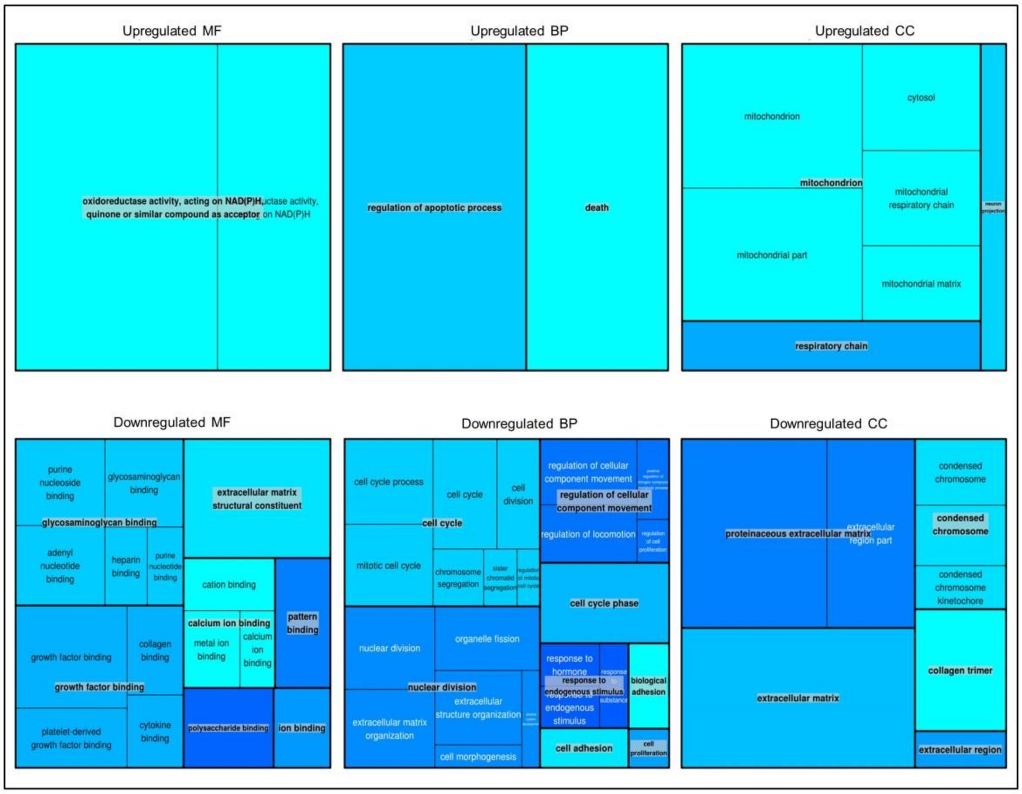

Supplementary Figure 3. Pathway/GO analysis in PTP4A1 silenced NHDF.. NGS data were analyzed with GO-Elite software. Images show pathway/GO term enrichment analysis for molecular functions (MF), biological processes (BP) and cellular compartments (CC) in 3 different NHDF lines treated with PTP4A1 ASO compared with the same lines treated with control ASO. Upper rows show pathways upregulated and lower rows show pathways downregulated in PTP4A1 silenced NHDF.

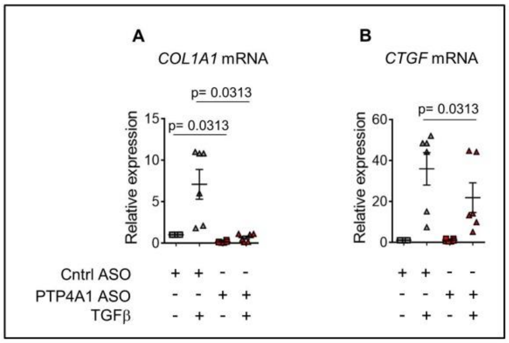

Supplementary Figure 4. PTP4A1 silencing downregulates TGFβ profibrotic genes. Mean±SEM of *COL1A1* (**A**) or *CTGF* (**B**) mRNA expression and 6 different NHDF lines treated with control or PTP4A1 ASO, stimulated with TGFβ1 and normalized to the same lines treated with control ASO. Wilcoxon test.

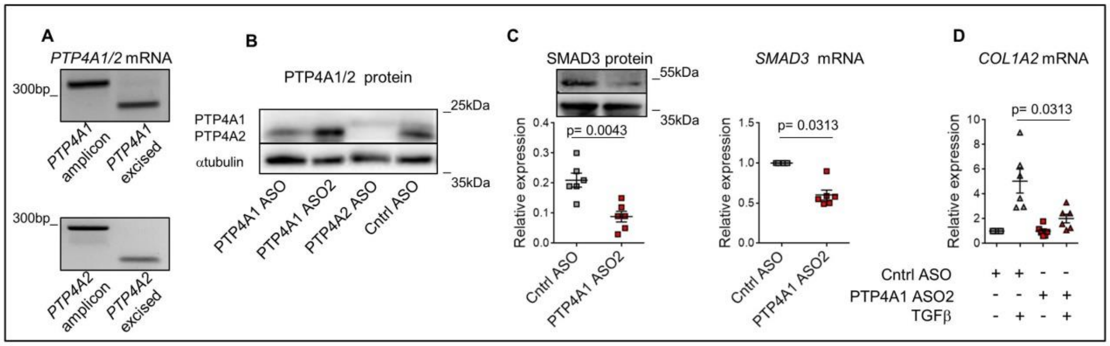

Supplementary Figure 5. PTP4A1 ASO (2) downregulates TGFβ profibrotic pathway. (**A**) Agarose gels with RT-PCR of *PTP4A1* (upper gel) and PTP4A2 (lower gel) mRNA from NHDF treated with control ASO (left lanes), PTP4A1 ASO2 or PTP4A2 ASO (right lanes). (**B**) Immunoblotting for PTP4A1/2 (upper bands) and GAPDH (lower bands) in NHDF treated with, PTP4A1 ASO, PTP4A1 ASO2, PTP4A2 ASO or control ASO. (**C**) Left graph shows mean±SEM of densitometric scan expression plus representative immunoblotting for SMAD3 (upper bands) normalized with GAPDH (lower bands) in 6 different NHDF lines treated with control ASO or PTP4A1 ASO2. Right graph shows mean±SEM of *SMAD3* mRNA expression measured in 6 different NHDF lines treated with PTP4A1 ASO2 and normalized to the same lines treated with control ASO. (**D**) Mean±SEM of *COL1A2* mRNA expression measured in 6 different NHDF lines treated with PTP4A1 ASO2, stimulated with TGFβ1 and normalized to the same lines treated with control ASO. Mann-Whitney test (**C** left) or Wilcoxon test (**C** right, **D**).

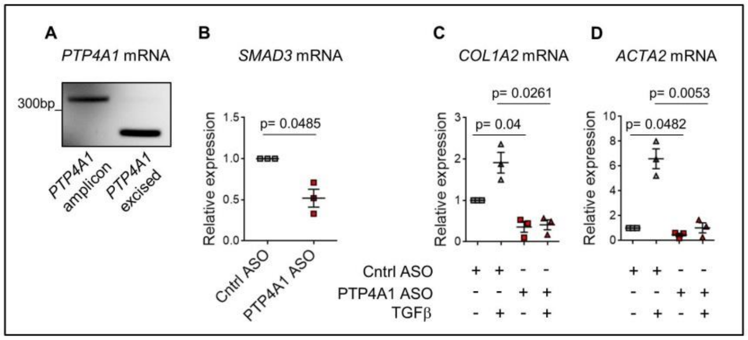

Supplementary Figure 6. PTP4A1 KO inhibits TGFβ signaling in lung fibroblasts. (**A**) Agarose gel with RT-PCR of *PTP4A1* mRNA from normal human lung fibroblasts (NHLF) treated with control ASO (left lane) or with anti-PTP4A1 ASO (right lane). (**B**) Mean±SEM of *SMAD3* mRNA expression measured in 3 different lines of NHLF treated with anti-PTP4A1 ASO and normalized to the same lines treated with control ASO. (**C**, **D**) Mean±SEM of *COL1A2* and *ACTA2* mRNA expression measured in 3 different NHLF lines treated with PTP4A1 ASO, stimulated with TGFβ1 and normalized to the same lines treated with control ASO. Paired t-test.

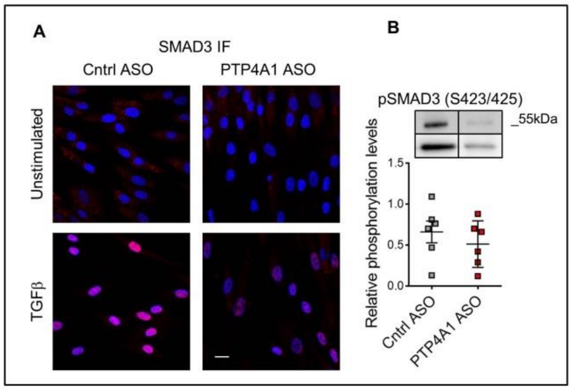

Supplementary Figure 7. PTP4A1 influences SMAD3 localization. (**A**) SMAD3 immunofluorescence (red signal) in NHDF treated with control ASO or PTP4A1 ASO and then stimulated with TGFβ1. Hoechst staining was used to visualize nuclei (blue signal). (**B**) Mean±SEM of densitometric scan expression plus representative immunoblotting for pSMAD3 (S423/425) (upper bands) normalized to SMAD3 (lower bands) in 6 different NHDF lines treated with control ASO or PTP4A1 ASO and then stimulated with TGFβ1. Images are from different lanes on the same membrane. Scale bar, 10 µm.

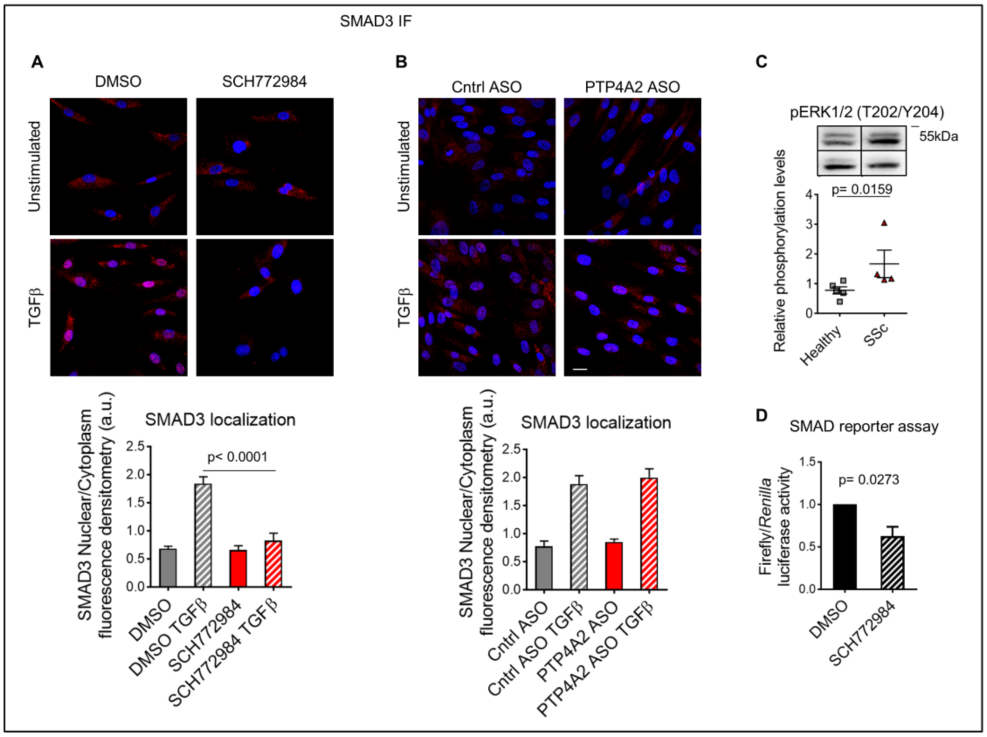

Supplementary Figure 8. ERK inhibition and PTP4A2 silencing effects on SMAD3. Top panels: SMAD3 immunofluorescence (red signal) in NHDF treated with SCH772984 ERK inhibitor (**A**) or PTP4A2 ASO (**B**) and stimulated with TGFβ. Hoechst staining was used to visualize nuclei (blue signal). Lower panels show mean±SEM of the ratio between SMAD3 nuclear and cytoplasmatic fluorescence densitometry in 3 NHDF lines. (**C**) Mean±SEM of densitometric scan expression plus representative immunoblotting for pERK1/2 (T202/Y204) (upper bands) normalized with ERK2 (lower bands) in 5 healthy or 4 SSc diffuse HDF lines. Images are from distinct membranes. (**D**) Mean±SEM of relative ratio of firefly/*Renilla* luciferase signal of HEK 293T treated with DMSO or SCH772984 ERK inhibitor together with a firefly luciferase SMAD reporter and a control luciferase *Renilla* vector. Cells were stimulated with TGFβ. Graph is representative of 3 biological replicates. Mann-Whitney test (**A**, **B** and **C**) and Welch's t-test (**D**). Scale bar, 10 µm.

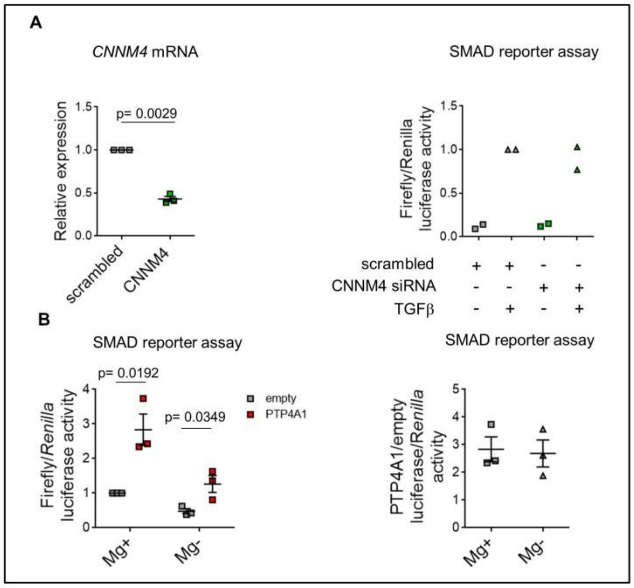

Supplementary Figure 9. Magnesium does not influence TGFβ-SMAD3 signaling. (**A**) Left graph shows mean±SEM of *CNNM4* mRNA expression measured by qPCR in HEK 293T cells treated with anti-*CNNM4* siRNA and normalized to cells treated with scrambled siRNA. Right graph shows relative ratio of firefly/*Renilla* luciferase signal of HEK 293T cells co-transfected with human scramble or anti-*CNNM4* siRNA together with a firefly luciferase SMAD reporter and a control luciferase *Renilla* vector. Cells were stimulated with TGFβ. Graphs are representative of 3 and 2 independent experiments. (**B**) Left graph shows mean±SEM of relative ratio of firefly/*Renilla* luciferase signal of HEK 293T cells co-transfected with human HA-tagged PTP4A1 WT –encoding pCDNA4 vector together with a firefly luciferase SMAD reporter and a control luciferase *Renilla* vector. Cells were incubated in normal or Mg starved media and stimulated with TGFβ. Graph is representative of 3 independent experiments. Right graph shows mean±SEM ratio of PTP4A1/empty firefly/Renilla luciferase signal between cells transfected with PTP4A1 or empty vectors. Paired t-test.

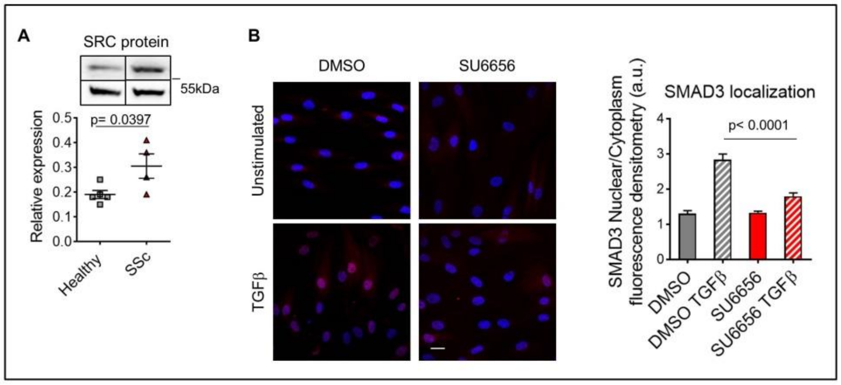

Supplementary Figure 10. SRC levels in SSc fibroblasts and SRC inhibition effects on SMAD3. (**A**) Mean±SEM of densitometric scan expression plus representative immunoblotting for SRC (upper bands) normalized with tubulin (lower bands) in 5 healthy or 4 SSc diffuse HDF lines. Images are from distinct membranes. (**B**) Images on the left show SMAD3 immunofluorescence (red signal) in NHDF treated with SU6656 SRC inhibitor and stimulated with TGFβ. Hoechst staining was used to visualize nuclei (blue signal). Right graph shows mean±SEM of the ratio between SMAD3 nuclear and cytoplasmatic fluorescence densitometry in 3 NHDF lines. Mann-Whitney test. Scale bar, 10 μm.

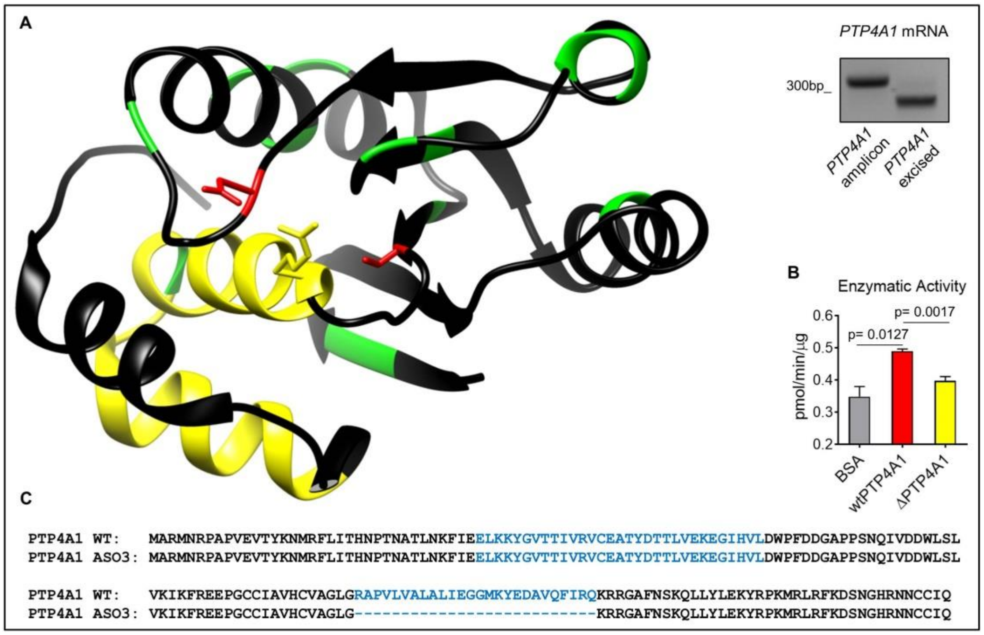

Supplementary Figure 11. Phosphatase dead PTP4A1 ASO (3) product (ΔPTP4A1). (**A**) Image shows, on the right, agarose gel with RT-PCR of PTP4A1 mRNA from NHDF treated with control ASO (left lanes) or PTP4A1 ASO3 (right lanes) and, on the left, the 3D structure of PTP4A1 from PDB database (1X24). Yellow-highlighted secondary structure shows the region where PTP4A1 ASO3 eliminates exon 4, between residues G109 and K136. Critical amino acids for catalysis are shown in red (D72, C104) or yellow (R110). Residues not shared with PTP4A2 are highlighted in green on the secondary structure. (**B**) Mean±SEM of BSA, wtPTP4A1 or Δ110-135PTP4A1 (ΔPTP4A1) specific activity with p-Nitrophenyl phosphate substrate. (**C**) PTP4A1 WT and PTP4A1 ASO3 product complete aa sequences. Exons 1, 3 and 5 are represented in black and exons 2 and 4 in blue. Welch's t-test.

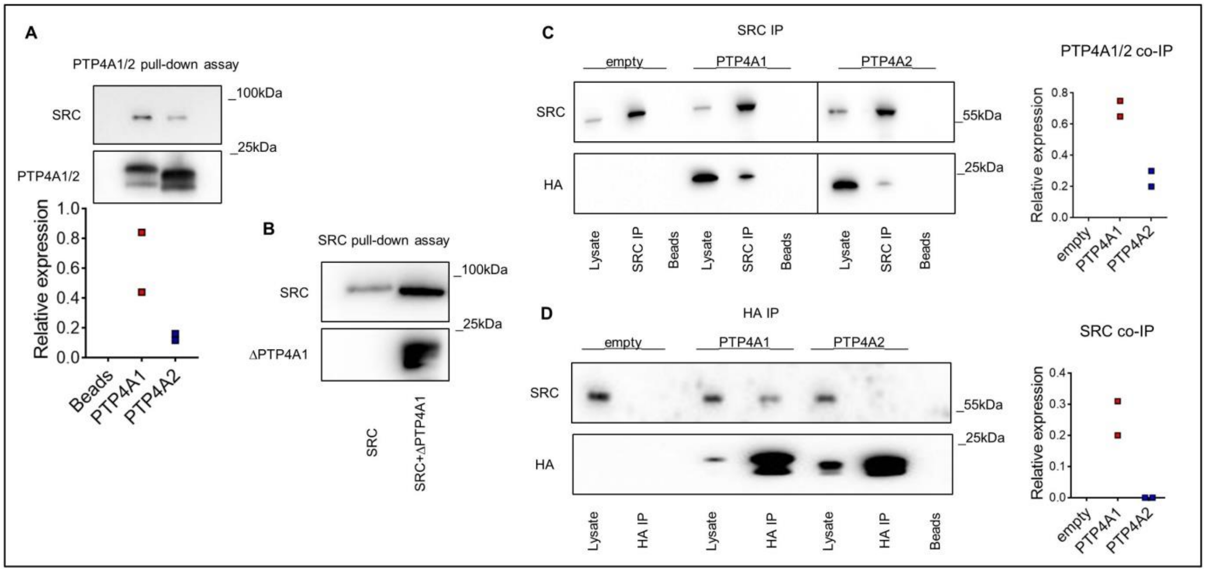

Supplementary Figure 12. PTP4A1 co-precipitates with SRC in HEK 293T cells. (**A**) Densitometric scan expression plus representative immunoblotting for SRC (upper bands) normalized to PTP4A1/2 (lower bands) in 2 different *in vitro* His-tagged PTP4A1/2 pull-down assay in presence of SRC. (**B**) Immunoblotting for SRC (upper bands) and ΔPTP4A1 (lower bands) in an in vitro SRC pull-down assay in presence of ΔPTP4A1. The image is representative of 2 independent experiments. (**C-D**) Images on the left show immunoblotting for SRC (upper bands) and HA (lower bands) in HEK 293T cells co-transfected with empty vector, HA-tagged PTP4A1 C104S (**C**) or WT (**D**) and PTP4A2 C101S (**C**) or WT (**D**) -encoding pCDNA4 vectors. Lysates were immunoprecipitated (IP) with anti-SRC (**C**) or anti-HA (**D**) antibodies. Graphs on the right show densitometric scan of HA-tagged protein co-IP normalized to SRC IP (**C**) or SRC protein co-IP normalized to HA IP (**D**). Graphs are representative of 2 independent experiments.

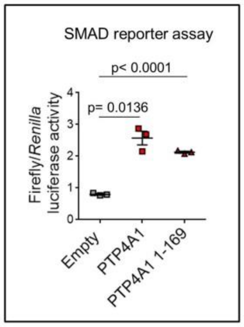

Supplementary Figure 13. SMAD reporter with PTP4A1 1-169. Mean±SEM of relative ratio of firefly/*Renilla* luciferase signal of HEK 293T cells co-transfected with human HA-tagged PTP4A1 WT or PTP4A1 1-169 (lacking membrane association) –encoding pCDNA4 vector together with a firefly luciferase SMAD reporter and a control luciferase *Renilla* vector. Cells were stimulated with TGFβ. Welch's t-test of 3 biological replicates.

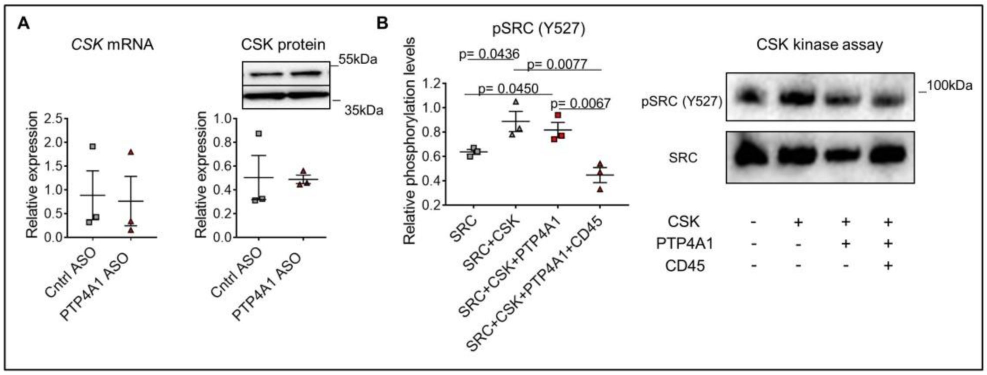

Supplementary Figure 14. PTP4A1 does not influence CSK expression or pSRC (Y527). (**A**) Left graph shows mean±SEM of *CSK* mRNA expression measured in 3 different NHDF lines treated with PTP4A1 ASO and normalized to same lines treated with control ASO. Right graphs show mean±SEM of densitometric scan expression plus representative immunoblotting for CSK (upper bands) normalized to GAPDH (lower bands) in 3 different NHDF lines treated with control ASO or PTP4A1 ASO. (**B**) Left graph shows mean±SEM of densitometric scan expression plus representative immunoblotting (right graph) for pSRC (Y527) (upper bands) normalized to SRC (lower bands) in 3 different *in vitro* kinase assay carried out in presence of CSK, PTP4A1 and CD45. Welch's t-test.

Patient ID	Age at onset	Sex	Race	Skin involvement	Stage	mRSS score	Immunosuppressants	Experimental Use
**American cohort**								
**1**	40	F	Caucasian	Diffuse	Early	27	No	mRNA
**2**	47	F	Caucasian	Diffuse	Early	30	No	mRNA
**3**	42	F	Caucasian	Diffuse	Early	29	No	mRNA/proteins
**4**	44	F	Caucasian	Diffuse	Early	14	Yes (MTX)	mRNA
**5**	47	F	Caucasian	Diffuse	Early	32	No	mRNA/proteins
**6**	38	M	Caucasian	Diffuse	Early	35	No	mRNA
**7**	49	M	Caucasian	Diffuse	Early	18	No	mRNA/proteins
**8**	61	M	Caucasian	Diffuse	Early	28	Yes (MMF)	mRNA/proteins
**9**	37	F	Caucasian	Diffuse	N/A	20	No	mRNA
**10**	45	F	Caucasian	Diffuse	N/A	15	No	mRNA
**11**	58	F	Caucasian	Diffuse	N/A	40	No	mRNA
**12**	21	F	Caucasian	Diffuse	N/A	26	No	mRNA
**13**	75	F	Caucasian	Diffuse	N/A	17	No	mRNA
**14**	44	F	Caucasian	Diffuse	Late	32	Yes	Immunofluorescence
**15**	56	F	African American	Diffuse	Early	44	No	Immunofluorescence
**16**	61	M	Caucasian	Diffuse	Early	26	No	Immunofluorescence
**17**	54	F	Caucasian	Diffuse	Late	19	No	Immunofluorescence
**18**	52	M	Caucasian	Diffuse	Early	25	No	Immunofluorescence
**19**	52	F	Caucasian	Diffuse	Late	6	Yes	Immunofluorescence
**20**	31	M	African American	Diffuse	Late	10	No	Immunofluorescence
**21**	50	F	Caucasian	Diffuse	Late	14	Yes	Immunofluorescence
**22**	61	M	African American	Diffuse	Early	31	No	Immunofluorescence
**23**	52	F	Caucasian	Diffuse	Early	20	No	Immunofluorescence
**24**	45	M	Caucasian	Diffuse	Late	23	No	Immunofluorescence
**25**	29	F	Caucasian	Limited	Late	2	Yes	Immunofluorescence
**26**	36	F	Caucasian	Limited	Late	7	No	Immunofluorescence
**27**	37	F	Caucasian	Limited	Late	3	No	Immunofluorescence
**28**	54	F	Caucasian	Limited	Late	2	No	Immunofluorescence
**29**	37	F	Caucasian	Limited	Late	4	No	Immunofluorescence
**30**	42	F	Caucasian	Limited	Late	2	No	Immunofluorescence
**Italian cohort**								
**1**	44	F	Caucasian	Diffuse	Early	11	No	Immunofluorescence
**2**	21	F	Caucasian	Diffuse	Early	12	No	Immunofluorescence
**3**	30	F	Caucasian	Diffuse	Early	7	No	Immunofluorescence
**4**	37	F	Caucasian	Diffuse	Early	10	No	Immunofluorescence
**5**	19	M	Caucasian	Diffuse	Early	10	No	Immunofluorescence
**6**	41	F	Caucasian	Diffuse	Late	17	No	Immunofluorescence
**7**	21	F	Caucasian	Diffuse	Late	14	No	Immunofluorescence
**8**	24	F	Caucasian	Diffuse	Late	13	No	Immunofluorescence
**9**	28	F	Caucasian	Diffuse	Late	13	No	Immunofluorescence
**10**	30	F	Caucasian	Diffuse	Late	11	No	Immunofluorescence

Supplementary Table 1. Clinical features of SSc patients.
Early disease stage <3 years from first non-Raynaud manifestation
mRSS score: Modified Rodnan Skin Score (1 to 51)
MMF: Mycophenolate mofetil
MTX: Methotrexate
N/A: Not available

Full unedited gel for Figure 1B
PTP4A1/2 expression in Healthy donors

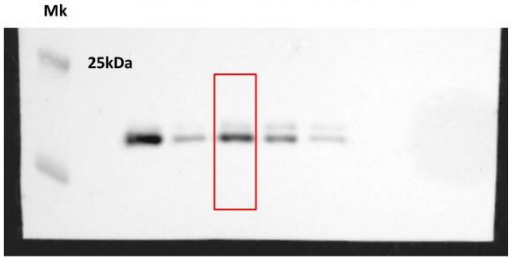

Full unedited gel for Figure 1B
PTP4A1/2 expression in SSc donors

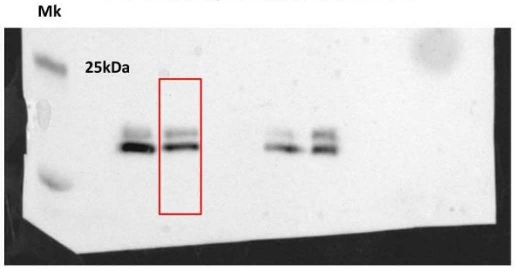

Full unedited gel for Figure 1B
Tubulin expression in Healthy donors

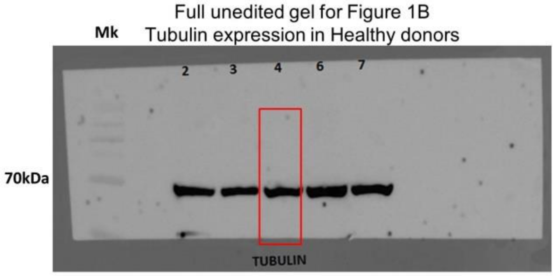

Full unedited gel for Figure 1B
Tubulin expression in SSc donors

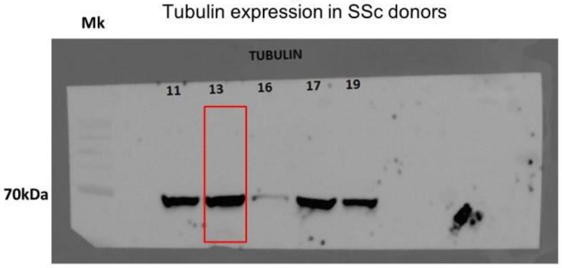

Full unedited gel for Figure 2A
PTP4A1 mRNA

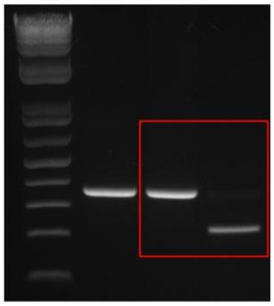

Full unedited gel for Figure 2D
SMAD3 expression

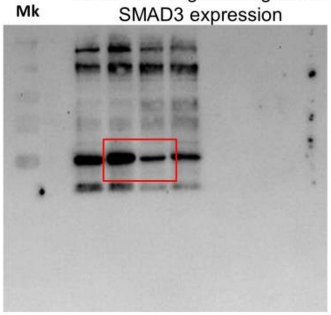

Full unedited gel for Figure 2D
GAPDH expression

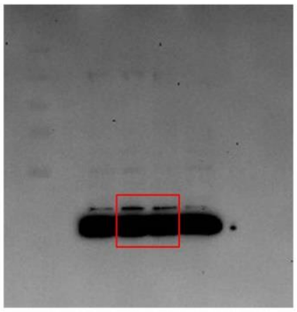

Full unedited gel for Figure 2E
αSMA expression

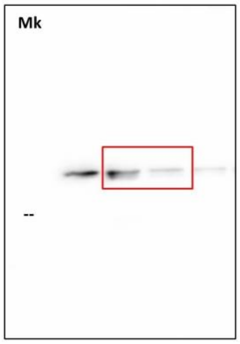

Full unedited gel for Figure 2E
GAPDH expression

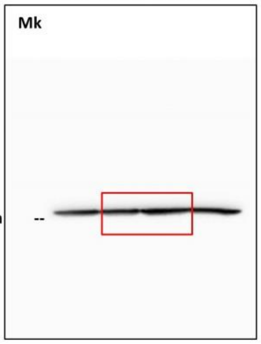

Full unedited gel for Figure 3g
SMAD3 expression in Cntrl or PTP4A2 ASO

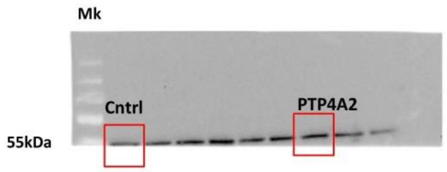

Full unedited gel for Figure 3g
GAPDH expression in Cntrl or PTP4A2 ASO

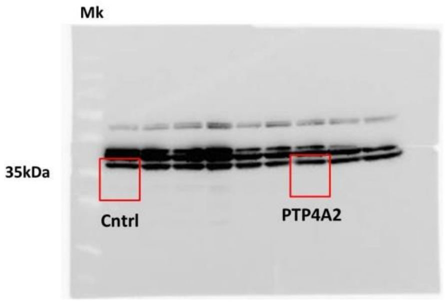

Full unedited gel for Figure 5D
pERK1/2 (T202/Y204) expression

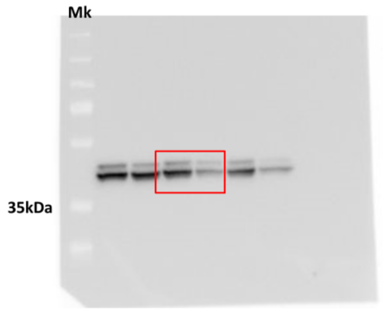

Full unedited gel for Figure 5E
pERK1/2 (T202/Y204) expression in Cntrl or PTP4A2 ASO

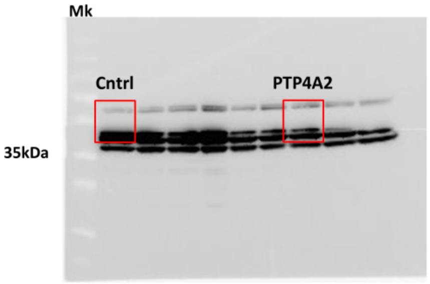

Full unedited gel for Figure 5E
GAPDH and PTP4A1/2 expression

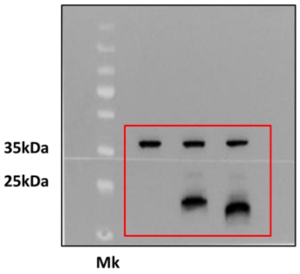

Full unedited gel for Figure 5D
ERK2 expression

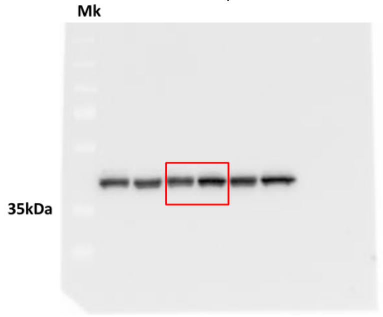

Full unedited gel for Figure 5E
ERK2 expression in Cntrl or PTP4A2 ASO

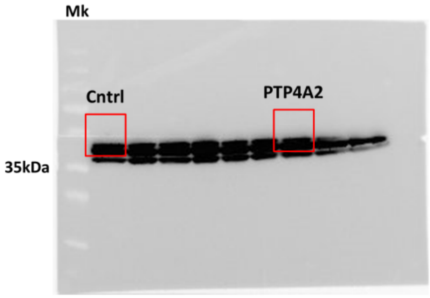

Full unedited gel for Figure 6A
pMEK1/2 (S217/221) expression
in Cntrl or PTP4A1 ASO

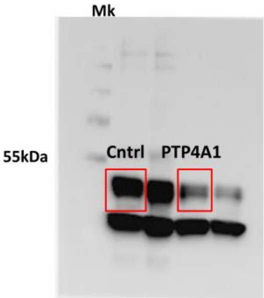

Full unedited gel for Figure 6B
pRAF (S259) expression

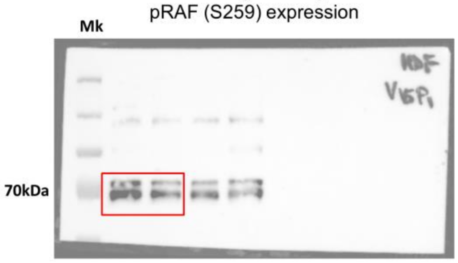

Full unedited gel for Figure 6C
SRC expression

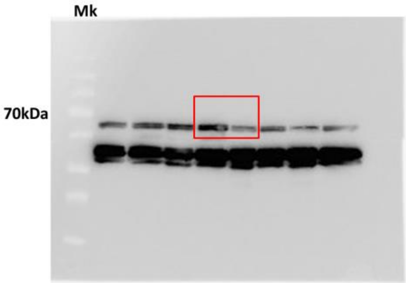

Full unedited gel for Figure 6A
MEK1/2 expression in Cntrl or PTP4A1 ASO

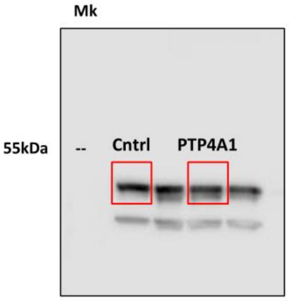

Full unedited gel for Figure 6B
GAPDH expression

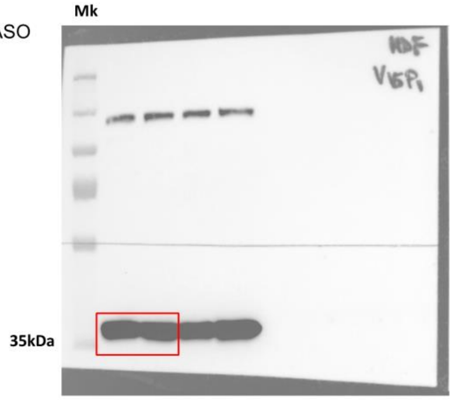

Full unedited gel for Figure 6C
GAPDH expression

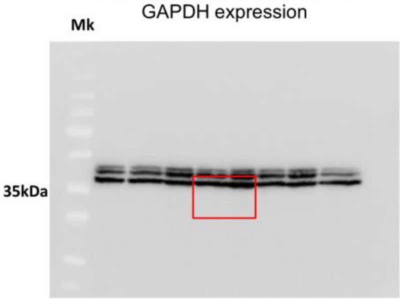

Full unedited gel for Figure 6D
SRC expression in Cntrl or PTP4A2 ASO

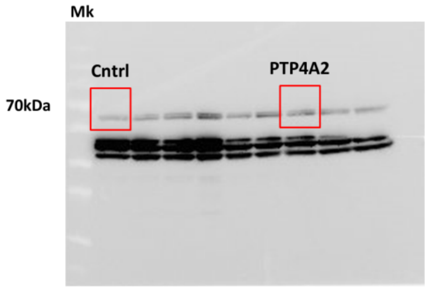

Full unedited gel for Figure 6E
pSRC (Y416) expression

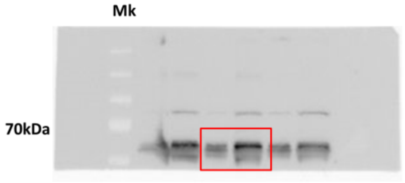

Full unedited gel for Figure 6D
GAPDH expression in Cntrl or PTP4A2 ASO

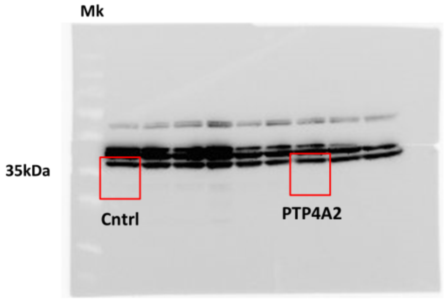

Full unedited gel for Figure 6E
SRC expression

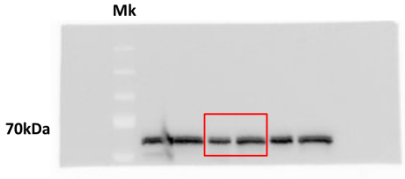

Full unedited gel for Figure 6E
pSRC (Y527) expression

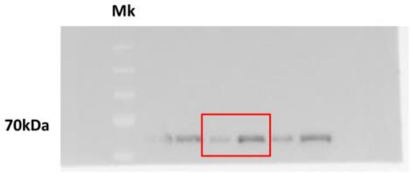

Full unedited gel for Figure 6F
pPLCg (Y783) expression

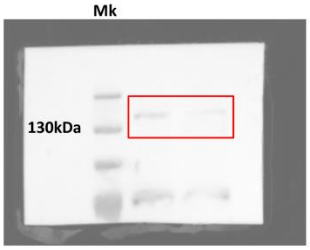

Full unedited gel for Figure 6E
SRC expression

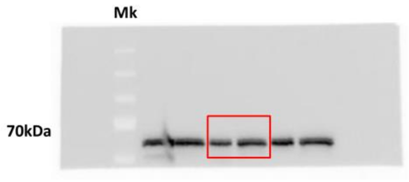

Full unedited gel for Figure 6F
GAPDH expression

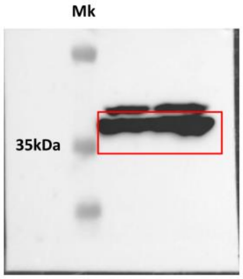

Full unedited gel for Figure 7B
SMAD3 expression

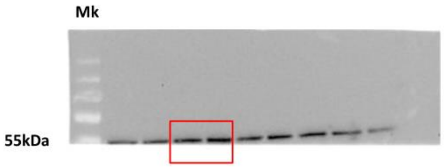

Full unedited gel for Figure 7C
SRC expression

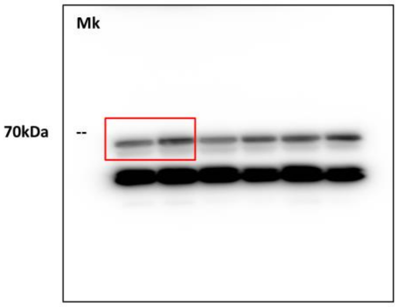

Full unedited gel for Figure 7D
pERK1/2 (T202/Y204) expression

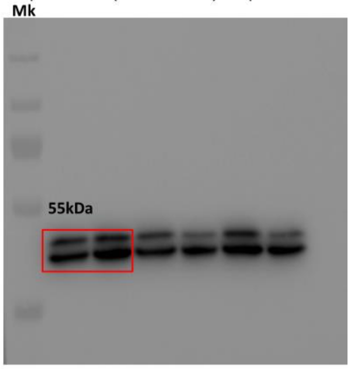

Full unedited gel for Figure 7B
GAPDH expression

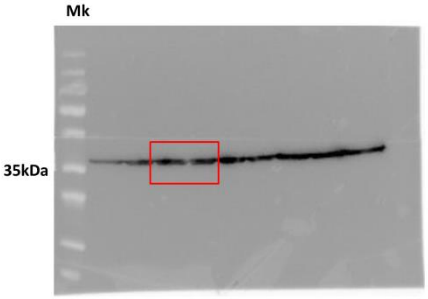

Full unedited gel for Figure 7C
Tubulin expression

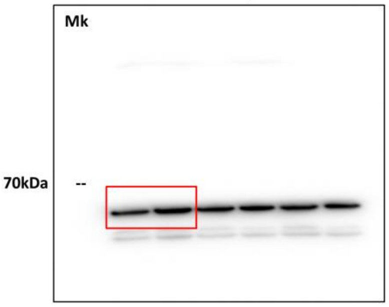

Full unedited gel for Figure 7D
Tubulin expression

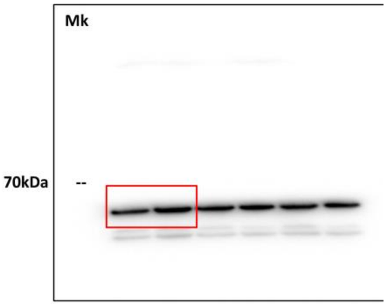

Full unedited gel for Figure 7E
pSRC (Y416) expression

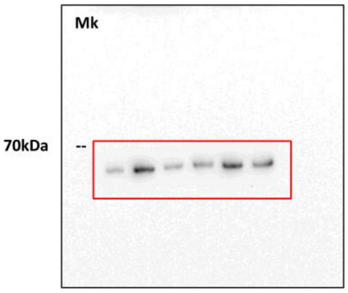

Full unedited gel for Figure 7F
PTP4A1/2 expression

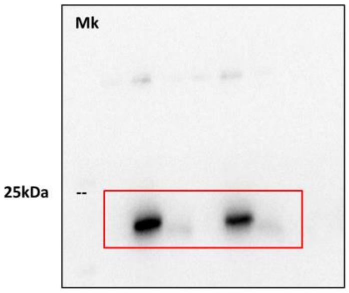

Full unedited gel for Figure 7G
pSRC (Y416) expression

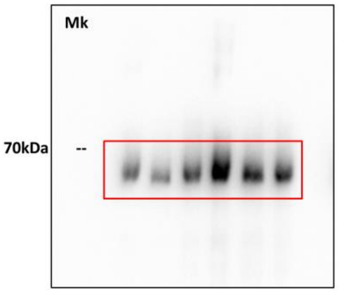

Full unedited gel for Figure 7E
SRC expression

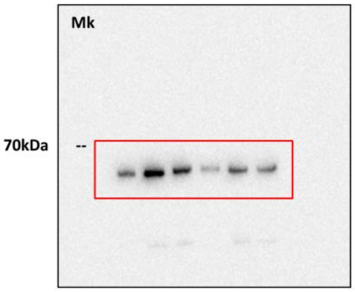

Full unedited gel for Figure 7F
SRC expression

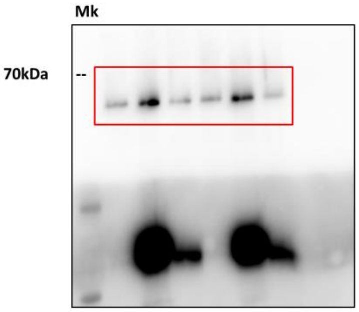

Full unedited gel for Figure 7G
SRC expression

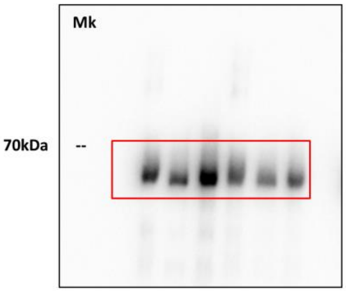

Full unedited gel for Supplementary Figure 5A
PTP4A1 mRNA

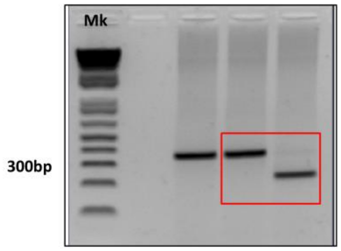

Full unedited gel for Supplementary Figure 5B
PTP4A1/2 expression

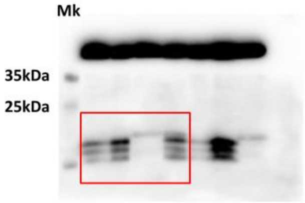

Full unedited gel for Supplementary Figure 5A
PTP4A2 mRNA

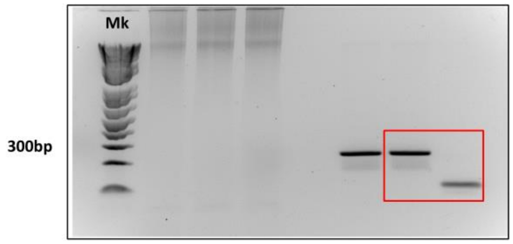

Full unedited gel for Supplementary Figure 5B
αtubulin expression

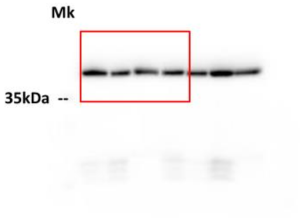

Full unedited gel for Supplementary Figure 5C
SMAD3 expression in Cntrl or PTP4A1 ASO

55kDa

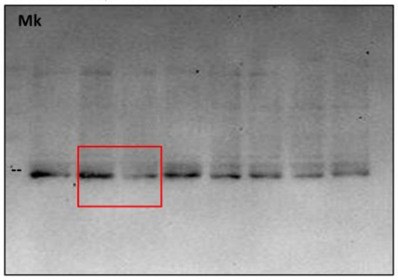

Full unedited gel for Supplementary Figure 5C
GAPDH expression in Cntrl or PTP4A1 ASO

55kDa

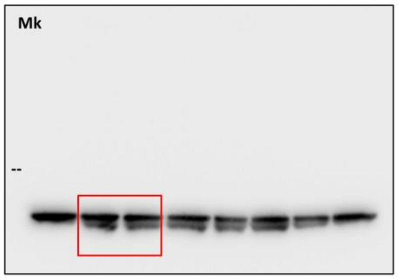

Full unedited gel for Supplementary Figure 6A
PTP4A1 mRNA

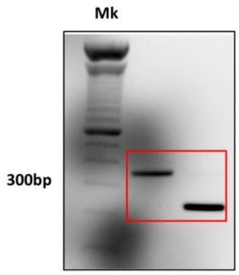

Full unedited gel for Supplementary Figure 7B
pSMAD3 (S423/425) expression in Cntrl or PTP4A1 ASO

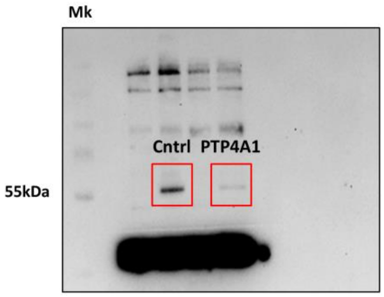

Full unedited gel for Supplementary Figure 7B
SMAD3 expression in Cntrl or PTP4A1 ASO

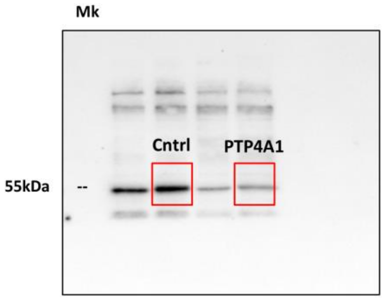

Full unedited gel for Supplementary Figure 8C
pERK1/2 (T202/Y204) expression in healthy dermal fibroblasts

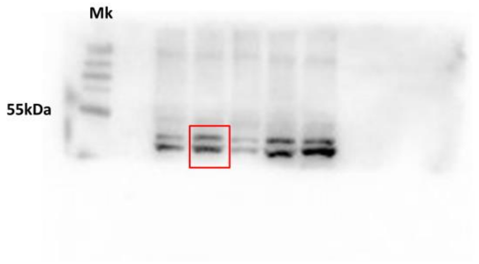

Full unedited gel for Supplementary Figure 8C
pERK1/2 (T202/Y204) expression in SSc dermal fibroblasts

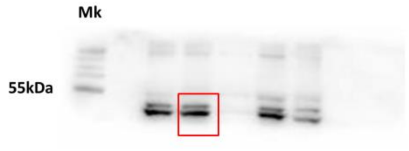

Full unedited gel for Supplementary Figure 8C
ERK2 expression in healthy dermal fibroblasts

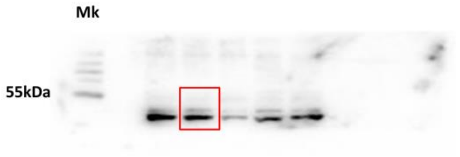

Full unedited gel for Supplementary Figure 8C
ERK2 expression in SSc dermal fibroblasts

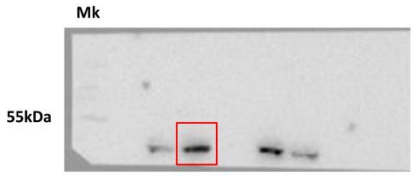

Full unedited gel for Supplementary Figure 10A
SRC expression in healthy dermal fibroblasts

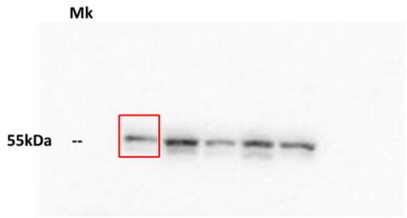

Full unedited gel for Supplementary Figure 10A
SRC expression in SSc dermal fibroblasts

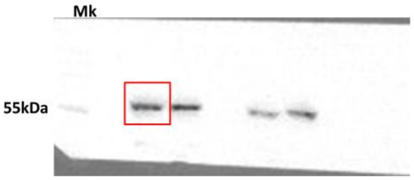

Full unedited gel for Supplementary Figure 10A
Tubulin expression in healthy dermal fibroblasts

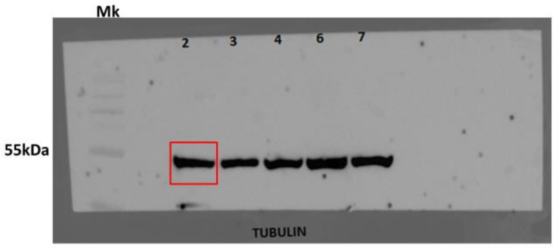

Full unedited gel for Supplementary Figure 10A
Tubulin expression in SSc dermal fibroblasts

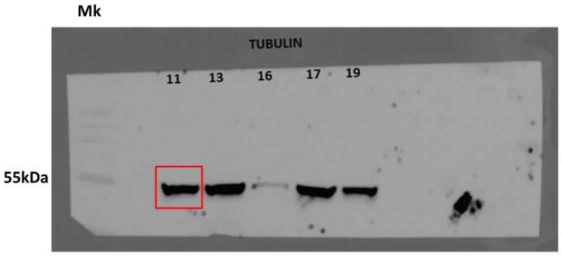

Full unedited gel for Supplementary Figure 11A *PTP4A1* mRNA

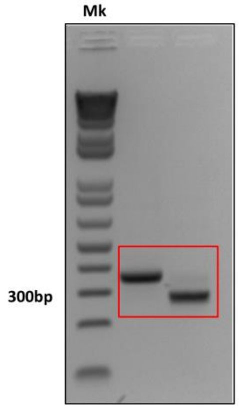

Full unedited gel for Supplementary Figure 12A SRC expression on PTP4A1/2 pull-down assay

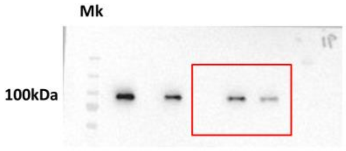

Full unedited gel for Supplementary Figure 12A HA expression on PTP4A1/2 pull-down assay

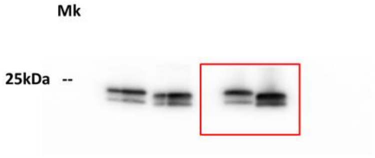

Full unedited gel for Supplementary Figure 12B SRC expression on ΔPTP4A1 pull-down assay

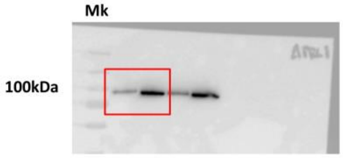

Full unedited gel for Supplementary Figure 12B HA expression on ΔPTP4A1 pull-down assay

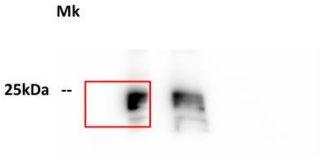

Full unedited gel for Supplementary Figure 12C
SRC expression on SRC IP

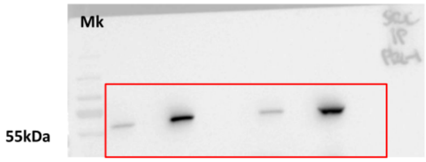

Full unedited gel for Supplementary Figure 12C
SRC expression on SRC IP

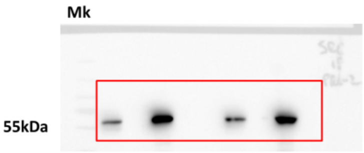

Full unedited gel for Supplementary Figure 12C
PTP4A1 expression on SRC IP

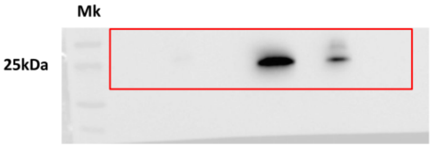

Full unedited gel for Supplementary Figure 12C
PTP4A2 expressionon SRC IP

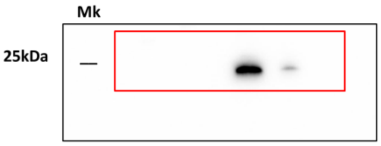

Full unedited gel for Supplementary Figure 12D
SRC expression on HA IP

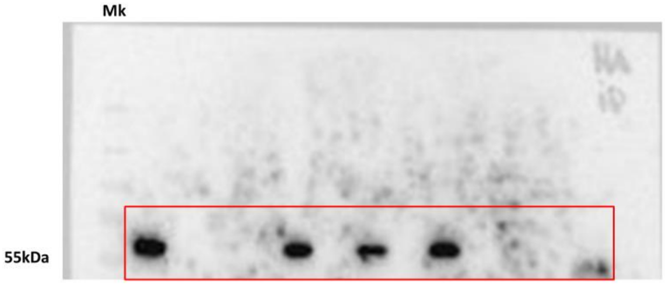

Full unedited gel for Supplementary Figure 12D
PTP4A1/2 expression on HA IP

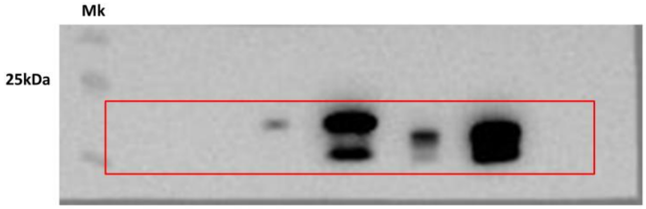

Full unedited gel for Supplementary Figure 14A
CSK expression in Cntrl or PTP4A1 ASO

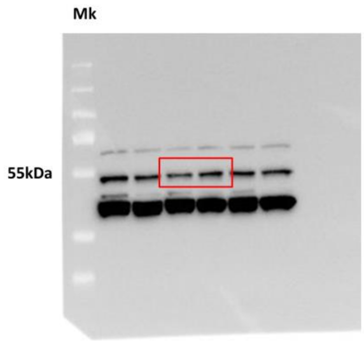

Full unedited gel for Supplementary Figure 14B
pSRC (Y527) expression

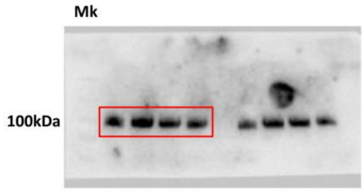

Full unedited gel for Supplementary Figure 14A
GAPDH expression in Cntrl or PTP4A1 ASO

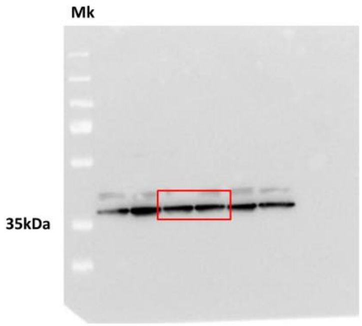

Full unedited gel for Supplementary Figure 14B
SRC expression

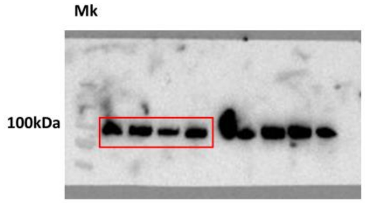

Supplement: Supplementary file 1 — Supplementary information [file 41467_2017_1168_MOESM1_ESM.pdf]
