# Supplementary material for: PTP4A1 promotes TGFβ signaling and fibrosis in systemic sclerosis
Source: Nat Commun. 2017 Oct 20;8:1060. doi: 10.1038/s41467-017-01168-1 (PMC5651906; doi:10.1038/s41467-017-01168-1)
Supplement: Supplementary file 3 — Description of Additional Supplementary Files [file 41467_2017_1168_MOESM3_ESM.pdf]

Description of Additional Supplementary Files

File Name: Supplementary Data 1
Description: Raw data for main figures

File Name: Supplementary Data 2
Description: Raw data for supplementary figures
